# Association of weight fluctuation with cardiovascular disease risk among initially obese adults

**DOI:** 10.1038/s41598-021-89666-7

**Published:** 2021-05-12

**Authors:** Seogsong Jeong, Seulggie Choi, Jooyoung Chang, Kyuwoong Kim, Sung Min Kim, Seo Yun Hwang, Joung Sik Son, Gyeongsil Lee, Sang Min Park

**Affiliations:** 1 Department of Biomedical Sciences, Seoul National University College of Medicine, 101 Daehak-ro, Jongno-gu, Seoul, 03080 Republic of Korea; 2 National Cancer Control Institute, National Cancer Center, Goyang-si, Gyeonggi-do Republic of Korea; 3 School of Health and Environmental Science, Korea University, Seoul, South Korea; 4 Department of Family Medicine, Korea University Guro Hospital, Seoul, 08308 South Korea; 5 Department of Family Medicine, Seoul National University Hospital, Seoul, Republic of Korea

**Keywords:** Cardiology, Health care

## Abstract

The association of fluctuations in body mass index with cardiovascular risk in long-term is not well understood. This study aimed to investigate cardiovascular outcomes of weight fluctuation. Total of 67,101 obese adults from the Korean National Health Insurance Service who received health examinations in three separate biennial periods were included. Participants were followed up from January 1, 2008 to the date of cardiovascular disease, death, or December 31, 2015, and categorized into 9 distinctive groups according to the BMI. Continuous weight gain showed an increased risk of overall cardiovascular disease (hazard ratio [HR], 2.36; *P* = 0.007), whereas weight loss after weight maintenance (HR, 0.91; *P* = 0.016) and weight maintenance after weight loss (HR, 0.91; *P* = 0.004) were ameliorative compared to the no weight change group. As for coronary heart disease, weight maintenance after weight gain was unfavorable (HR, 1.25; *P* = 0.004) while weight loss after weight maintenance (HR, 0.82; *P* < 0.001), weight cycling (HR, 0.83; *P* = 0.043), and weight maintenance after weight loss (HR, 0.88; *P* = 0.012) were beneficial. Weight maintenance after weight loss is beneficial for obese adults in terms of cardiovascular risks. In addition, weight loss is in part related to reduced risk of coronary heart disease despite weight cycling.

## Introduction

Cardiovascular disease (CVD), including coronary heart disease (CHD) and stroke, is the most common non-communicable disease worldwide, responsible for 17.8 million deaths in 2017^1^. In a recent literature, a positive association was reported between body mass index (BMI) and cardiovascular risk, including morbidity and mortality^2^. According to the World Health Organization (WHO) risk models derived with use of emerging risk factors collaboration data, 1.18 and 1.14 of main effect hazard ratios (HR) were found for men and women per 1 kg/m^2^ of BMI^3^. Although preventable, the burden of obesity has reached epidemic proportions globally, which was once only prevalent in high-income countries^4^. Therefore, comprehensive studies regarding relations of BMI and CVD are of global interest to reduce the future burden CVD.

On contrary, a previous meta-analysis concluded that overweight and moderate obesity are associated with lower mortality possibly due to cardioprotective metabolic effects^5,6^. As a number of researchers pointed that it may be due to inaccuracy of BMI for measurement of total adiposity because it is a height-normalized sum of fat mass and fat-free mass, effects of BMI on cardiovascular risk are controversial^7^. However, pathophysiology and hemodynamics of CVD supported that higher fat-free mass is responsible for higher circulating blood volume responsible for increases in the left ventricular stroke volume and the cardiac output, contributing to ventricular hypertrophy and enlargement, which are predisposing conditions of heart failure^8,9^. Therefore, BMI is still the most common anthropometric index used to predict CVD-related mortality^10^.

In recent years, weight loss maintenance was reported to have ameliorative effect to cardiovascular risk factors, including triglyceride and blood pressure, whereas BMI variability was found a risk factor for CVD risk^11–13^. However, effects of circumstances of weight per BMI maintenance on cardiovascular outcomes in obese population remain unclear. Hence, we conducted the present study to further explore the impact of weight maintenance status in morbidity and mortality of CVD in Korean adults with obesity aged 40 and over.

## Methods

### Study population

**Figure 1:**
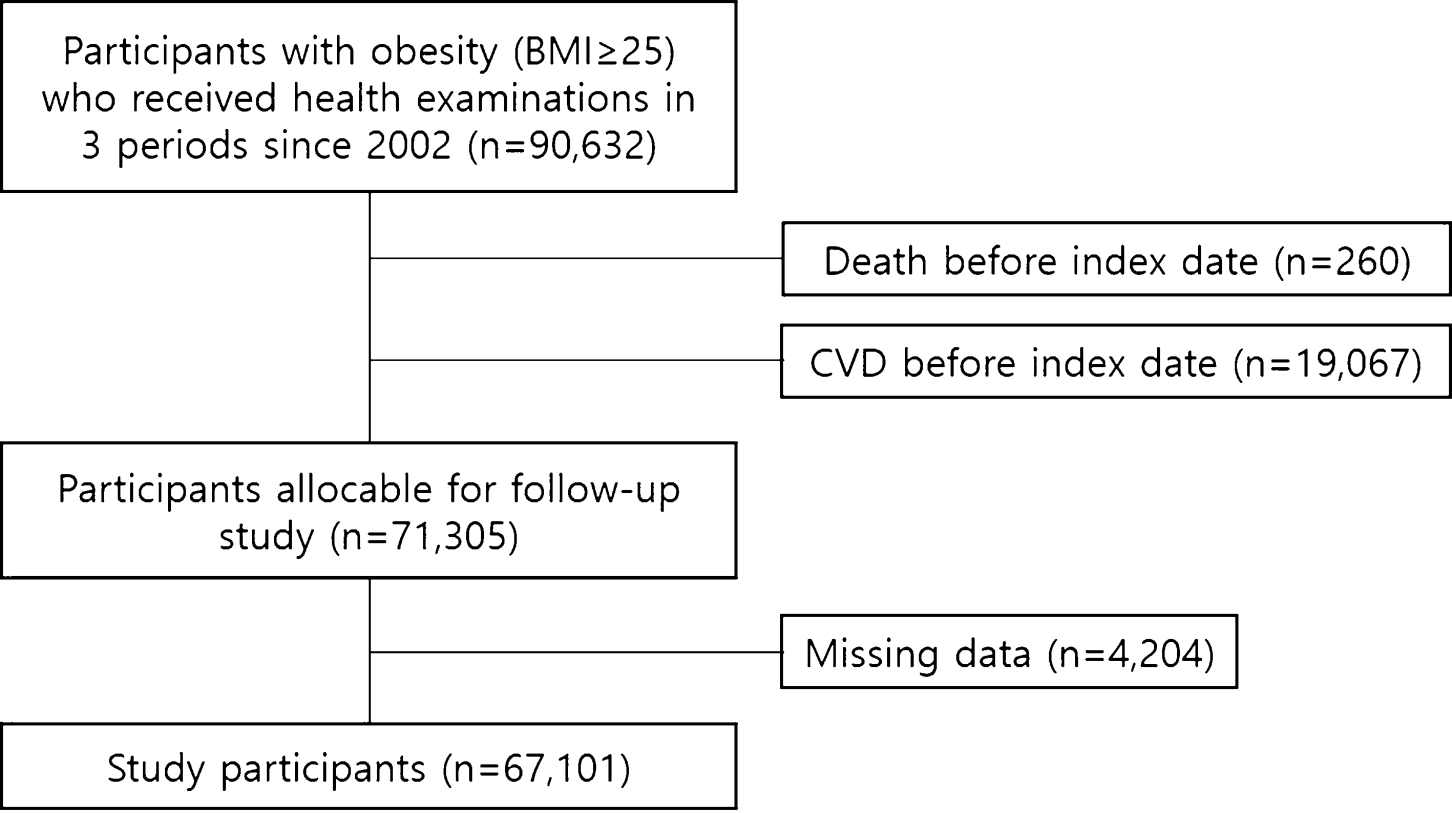
Participant inclusion flowchart.

The study participants were from the National Health Insurance Service-Health Screening Cohort (NHIS-HEALS) database, which provides mandatory health insurance for all Korean population^14^. Data on demographic characteristics, follow-up information, and health examination outcomes were merged with the claims data, which were anonymized according to the strict confidential guidelines as described previously^15^. A total of 90,632 participants with obesity (BMI ≥ 25 kg/m^2^; according to the Asia–Pacific classification) who received one or more health examinations in all time periods, including 2002 to 2003, 2004 to 2005, and 2006 to 2007, were identified (Fig. 1)^16^. Among them 260 and 19,067 participants were excluded from the analysis due to death and CVD before the index date, respectively. In addition, 4,204 participants were excluded due to missing data on covariates. Finally, 67,101 participants were enrolled for the analyses. This study adheres with the principles outlined in the Declaration of Helsinki. The Seoul National University Hospital Institutional Review Board approved this study (IRB number: X-1701-378-902). The requirement for informed consent from the participants was waived as the NHIS-HEALS database is anonymized according to strict confidentiality guidelines.

### BMI classification

All participants were stratified into 9 BMI maintenance status categories, including continuous weight gain (BMI change ≥ 2 kg/m^2^ at the 2nd period and additional BMI change ≥ 2 kg/m^2^ at the 3rd period), weight maintenance after weight gain (BMI change ≥ 2 kg/m^2^ at the 2nd period and BMI maintenance at the 3rd period), weight loss after weight gain (BMI change ≥ 2 kg/m^2^ at the 2nd period and BMI change ≤  − 2 kg/m^2^ at the 3nd period) weight gain after weight maintenance (BMI maintenance at the 2nd period and BMI change ≥ 2 kg/m^2^ at the 3rd period), no weight change (BMI maintenance at both 2nd and 3rd periods), weight loss after weight maintenance (BMI maintenance at the 2nd period and BMI change ≤  − 2 kg/m^2^ at the 3rd period), weight cycling (BMI change ≤  − 2 kg/m^2^ at the 2nd period and BMI change ≥ 2 kg/m^2^ at the 3rd period), weight maintenance after weight loss (BMI change ≤  − 2 kg/m^2^ at the 2nd period and BMI maintenance at the 3rd period), and continuous weight loss (BMI change ≤  − 2 kg/m^2^ at the 2nd period and additional BMI change ≤  − 2 kg/m^2^ at the 3rd period) groups, considering a BMI change of 2 kg/m^2^ as a large change (weight change of 4 kg; Supplementary Fig. 1). The term maintenance was defined as BMI change of less than 2 kg/m^2^.

### Definition of CVD and related outcome

CVD was defined using the Tenth Revision of International Classification of Diseases (ICD-10) codes from the WHO. Coronary heart disease (ICD-10 code, I20 to I25) and stroke (ICD-10 code, I60 to I69) were involved in CVD. CVD event was defined as cases with ≥ 3 hospital visits or ≥ 1 hospital admission with at least one of the above ICD-10 codes for CVD from the index date (1 January 2008). We defined CVD-related death as participants with a death date between 1 January 2008 and 31 December 2015 with CVD as the cause of death. Follow-up investigation started from 1 January 2008 and ended at the date of death or 31 December 2015 for all participants.

### Key variables for the adjustments

Following variables were considered for the adjustments: age (continuous; years), sex (categorical; men and women), household income (categorical; deciles), initial BMI (continuous; kg/m^2^), systolic blood pressure (continuous; mmHg), fasting serum glucose (FSG; continuous; mg/dL), total cholesterol (TC; continuous; mg/dL), aspartate aminotransferase (AST; continuous; IU/L), Charlson comorbidity index (continuous), smoking (categorical; never, previous, and current), alcohol consumption frequency (categorical; none, 1–6 days/week, and everyday), and exercise frequency (none, 1–6 days/week, and everyday).

### Statistical analysis

Continuous variables, including age, BMI, blood pressure, FSG, TC, AST, alanine aminotransferase (ALT), and γ-glutamyl transpeptidase (γ-GT) were presented with mean (standard deviation; SD), whereas categorical variables were described with n (%). The Cox proportional hazards regression model was applied for evaluation of time-dependent prognostic impact of BMI maintenance status, which was described using the hazard ratio (HR) with 95% confidence interval (CI). No weight change group was set as the reference group in the multivariable-adjusted Cox proportional hazards regression. Sensitivity analyses were performed by washing out 3 years of latent period since the index date. Subgroup analyses were performed after stratifying the participants according to age, sex, Charlson comorbidity index, smoking, alcohol consumption, and exercise. Changes in cardiometabolic risk factors were analyzed by the t-test, which were presented with mean (SD) and pooled *P* value. A *P* value of less than 0.05 was considered statistically significant. All statistical analyses were performed using the SAS software version 9.4 (SAS Institute Inc, Cary, NC).

## Results

### Participant characteristic

There were 1,659 participants with weight gain (BMI change ≥ 2 kg/m^2^), 59,975 participants with no weight change (|BMI change|< 2 kg/m^2^), and 5,467 participants with weight loss (BMI change ≤  − 2 kg/m^2^) at the 2nd period with mean ages of 51.3, 50.5, and 51.2 years, respectively (Table 1). Sex distribution was about 1:1 in weight gain and weight loss groups, whereas a high distribution of male participants (63.2%) was found to maintain BMI. Those who gained or maintained weight were more likely to have relatively lower baseline BMI (27.1 kg/m^2^ and 26.9 kg/m^2^) compared to the participants with weight loss (BMI, 28.1 kg/m^2^). The mean systolic and diastolic blood pressures were similar among the BMI groups. The proportion of Charlson comorbidity index ≥ 1 was higher in the gain (37.4%) and loss (35.5%) groups compared to the stable (33.2%) group, but the proportion of current smokers was highest in the stable group (24.5%). Approximately a half of the participants in the no weight change group showed no alcohol consumption and no exercise.

**Table 1:** Demographic characteristics of the obese adults according to the body mass index at second period (2004–2005).

Characteristic	Weight gain (n = 1,659)	No weight change (n = 59,975)	Weight loss (n = 5,467)
Age, years	51.3 (9.0)	50.5 (8.1)	51.2 (8.6)
**Sex, n (%)**			
Female	822 (49.5)	22,073 (36.8)	2,702 (49.4)
Male	837 (50.5)	37,902 (63.2)	2,765 (50.6)
Body mass index, kg/m^2^	27.1 (2.0)	26.9 (1.7)	28.1 (3.8)
Systolic blood pressure, mmHg	130.3 (17.8)	129.6 (17.1)	130.5 (17.7)
Diastolic blood pressure, mmHg	81.8 (11.5)	81.8 (11.4)	81.9 (11.5)
Fasting serum glucose, mg/dL	100.7 (40.4)	98.8 (31.6)	101.1 (35.9)
Total cholesterol, mg/dL	205.0 (39.3)	206.3 (38.2)	207.7 (39.7)
Aspartate aminotransferase, IU/L	28.3 (17.2)	28.2 (16.1)	28.9 (19.8)
Alanine aminotransferase, IU/L	29.8 (21.7)	31.2 (22.8)	31.1 (23.6)
γ-glutamyl transpeptidase, IU/L	42.0 (56.0)	44.0 (48.2)	41.5 (48.2)
**Charlson comorbidity index**			
0	1,038 (62.6)	40,082 (66.8)	3,524 (64.5)
1	427 (25.7)	14,123 (23.6)	1,332 (24.4)
≥ 2	194 (11.7)	5,770 (9.6)	611 (11.2)
**Smoking status, n (%)**			
Never	1,136 (68.5)	37,947 (63.3)	3,841 (70.3)
Previous	136 (8.2)	7,112 (11.2)	542 (9.9)
Current	387 (23.3)	14,916 (24.5)	1,084 (19.8)
**Alcohol consumption frequency, n (%)**			
None	937 (56.5)	29,750 (49.6)	3,110 (56.9)
1–6 days	666 (40.1)	28,148 (46.9)	2,157 (39.5)
Everyday	56 (3.4)	2,077 (3.5)	200 (3.7)
**Exercise frequency, n (%)**			
None	940 (56.7)	30,275 (50.5)	3,058 (55.9)
1–6 days	613 (36.9)	25,716 (42.9)	2,086 (38.2)
Everyday	106 (6.4)	3,984 (6.6)	323 (5.9)

Data are mean (standard deviation) unless indicated otherwise.

### Outcomes of weight gain, no change, and loss at 2nd period in terms of CVD and CVD-related death

We first simply evaluated whether weight gain, no change, or loss derives significantly different CVD-related outcomes (Supplementary Table 1). Unadjusted univariable analyses revealed that weight gain significantly reduces CVD-free survival (HR, 1.16; 95% CI, 1.05–1.27) and CHD-free survival (HR, 1.26; 95% CI, 1.10–1.44), but showed no significant impact in stroke and CVD-related death compared with the no weight change group. Similar results were observed after adjustments for age and sex. However, no significant risk was found for weight gain when further adjusted for household income, baseline BMI, systolic blood pressure, FSG, TC, and AST, whereas significant benefit was found for weight loss in terms of CVD- (HR, 0.92; 95% CI, 0.87–0.98; *P* = 0.007) and CHD-free survival (HR, 0.88; 95% CI, 0.81–0.96; *P* = 0.005). Collectively, one-dimensional weight change seems to significantly affect CVD and CHD, but not stroke.

### Effects of 9 BMI maintenance status categories for CVD

Subsequently, we sought to confirm whether morbidity and mortality of CVD are dependent on BMI maintenance status (Table 2). Compared to the no weight change group, continuous weight gain (HR, 2.36; 95% CI, 1.27–4.40; *P* = 0.007) revealed to be deteriorative for CVD-free survival. In contrast, weight loss after weight maintenance (HR, 0.91; 95% CI, 0.85–0.98; *P* = 0.016) and weight maintenance after weight loss (HR, 0.91; 95% CI, 0.85–0.97; *P* = 0.004) were protective against CVD, but not weight cycling (HR, 0.95; 95% CI, 0.85–1.06; *P* = 0.338) and continuous weight loss (HR, 0.95; 95% CI, 0.65–1.38; *P* = 0.772) groups, indicating that not all cases of weight loss is protective against CVD. Furthermore, significant disparities were found when CVD was stratified to CHD and stroke. Weight maintenance after weight gain significantly reduced CHD-free survival (HR, 1.25; 95% CI, 1.08–1.46; *P* = 0.004), whereas it was prolonged in weight loss after weight maintenance (HR, 0.82; 95% CI, 0.73–0.92; *P* < 0.001), weight cycling (HR, 0.83; 95% CI, 0.70–0.99; *P* = 0.043), weight maintenance after weight loss (HR, 0.88; 95% CI, 0.80–0.97; *P* = 0.012) groups. As for stroke, only continuous weight gain was significantly affective (HR, 2.77; 95% CI, 1.24–6.17; *P* = 0.013). In addition, there was no significant impact of BMI maintenance status in CVD-related death probably due to small number of CVD-related death.

**Table 2:** Effects of body mass index maintenance status at third period health examination on risk of cardiovascular disease, coronary heart disease, stroke, and cardiovascular disease-related death.

Outcome	Continuous weight gain (n = 18)	Weight maintenance after weight gain (n = 1,222)	Weight loss after weight gain (n = 419)	Weight gain after weight maintenance (n = 1,743)	No weight change (n = 55,023)	Weight loss after weight maintenance (n = 3,209)	Weight cycling (n = 1,253)	Weight maintenance after weight loss (n = 4,110)	Continuous weight loss (n = 104)
**CVD**									
Events (%)	10 (55.6)	326 (26.7)	111 (26.5)	430 (24.7)	13,141 (23.9)	754 (23.5)	311 (24.8)	968 (23.6)	27 (26.0)
Person-years	105	8,210	2,804	11,870	378,601	21,992	8,605	28,210	658
HR (95% CI)^a^	2.75 (1.24–6.13)	1.10 (0.95–1.27)	1.04 (0.81–1.34)	0.96 (0.84–1.10)	Reference	0.90 (0.82–1.00)	0.98 (0.85–1.14)	0.91 (0.83–0.99)	0.60 (0.31–1.16)
HR (95% CI)	2.36 (1.27–4.40)	1.08 (0.96–1.20)	1.03 (0.85–1.24)	0.98 (0.89–1.08)	Reference	0.91 (0.85–0.98)	0.95 (0.85–1.06)	0.91 (0.85–0.97)	0.95 (0.65–1.38)
*P* value	0.007	0.190	0.763	0.673	Reference	0.016	0.338	0.004	0.772
**CHD**									
Events (%)	4 (22.2)	172 (14.1)	52 (12.4)	191 (11.0)	6,190 (11.2)	304 (9.5)	124 (9.9)	434 (10.6)	12 (11.5)
Person-years	133	8,728	2,971	12,592	400,747	23,349	9,215	29,845	712
HR (95% CI)^a^	2.29 (0.74–7.10)	1.29 (1.06–1.58)	1.01 (0.69–1.47)	0.91 (0.75–1.11)	Reference	0.77 (0.66–0.90)	0.90 (0.71–1.13)	0.83 (0.72–0.95)	0.73 (0.30–1.76)
HR (95% CI)	1.73 (0.65–4.62)	1.25 (1.08–1.46)	1.09 (0.83–1.43)	0.97 (0.84–1.12)	Reference	0.82 (0.73–0.92)	0.83 (0.70–0.99)	0.88 (0.80–0.97)	0.94 (0.53–1.66)
*P* value	0.273	0.004	0.539	0.653	Reference	< 0.001	0.043	0.012	0.834
**Stroke**									
Events (%)	6 (33.3)	157 (12.8)	60 (14.3)	242 (13.9)	7,029 (12.8)	451 (14.1)	188 (15.0)	539 (13.1)	15 (14.4)
Person-years	115	8,958	3,043	12,777	406,405	23,424	9,121	30,193	715
HR (95% CI)^a^	2.47 (0.80–7.65)	0.94 (0.77–1.15)	1.08 (0.79–1.49)	1.00 (0.85–1.18)	Reference	1.04 (0.92–1.17)	1.05 (0.87–1.26)	1.00 (0.90–1.12)	0.42 (0.16–1.13)
HR (95% CI)	2.77 (1.24–6.17)	0.91 (0.77–1.06)	0.98 (0.76–1.26)	0.98 (0.87–1.12)	Reference	1.00 (0.91–1.10)	1.05 (0.91–1.21)	0.95 (0.87–1.04)	0.93 (0.56–1.54)
*P* value	0.013	0.225	0.867	0.812	Reference	0.972	0.544	0.226	0.774
**CVD**–**related death**									
Events (%)	0 (0)	2 (0.2)	3 (0.7)	10 (0.6)	218 (0.4)	13 (0.4)	5 (0.4)	28 (0.7)	2 (1.9)
Person-years	144	9,593	3,247	13,719	434,017	25,137	9,854	32,187	780
HR (95% CI)^a^	NA	0.20 (0.03–1.42)	1.19 (0.29–4.81)	0.89 (0.39–2.01)	Reference	0.74 (0.38–1.44)	0.98 (0.40–2.39)	1.25 (0.78–2.02)	1.77 (0.25–12.8)
HR (95% CI)	NA	0.32 (0.08–1.29)	1.44 (0.46–4.50)	1.22 (0.65–2.31)	Reference	0.85 (0.49–1.50)	0.78 (0.32–1.91)	1.47 (0.98–2.20)	2.85 (0.70–11.6)
*P* value	0.979	0.110	0.534	0.538	Reference	0.579	0.592	0.062	0.143

HR calculated by Cox proportional hazards regression analysis after adjustments for age, sex, household income, initial body mass index, systolic blood pressure, fasting serum glucose, total cholesterol, aspartate aminotransferase, Charlson comorbidity index, smoking, alcohol consumption, and exercise frequency. ^a^3 years of latent period washed out for sensitivity analysis. Acronyms: CVD, cardiovascular disease; HR, hazard ratio; CI, confidence interval; CHD, coronary heart disease; NA, not applicable.

### Benefits of weight maintenance after weight loss in terms of cardiovascular risks compared to weight cycling, weight loss after weight maintenance, and no weight change

Since significant protective effects were found in weight maintenance after weight loss against CVD and CHD, we assessed whether there is a significant risk of weight cycling, weight loss after weight maintenance, and no weight change (Table 3). Unexpectedly, no significant risk was found for weight cycling and weight loss after weight maintenance, unlike no weight change that revealed significantly increased HR for both CVD (1.10; 95% CI, 1.03–1.18; *P* = 0.005) and CHD (1.13; 95% CI, 1.03–1.25; *P* = 0.014), indicating that weight loss attempts are more favorable BMI maintenance status compared to no weight change.

**Table 3:** Effects of weight loss maintenance on cardiovascular risk compared to those with weight loss-weight gain, weight stable-weight loss, and weight stable-weight maintenance groups.

Outcome	Weight maintenance after weight loss	Weight cycling	Weight loss after weight maintenance	No weight change
**CVD**				
Events (%)	968 (23.6)	311 (24.8)	754 (23.5)	13,141 (23.9)
Person-years	28,210	8,605	21,992	378,601
HR (95% CI)	Reference	1.04 (0.92–1.18)	1.01 (0.91–1.11)	**1.10 (1.03–1.18)**
*P* value	Reference	0.535	0.921	**0.005**
**Coronary heart disease**				
Events (%)	434 (10.6)	124 (9.9)	304 (9.5)	6,190 (11.2)
Person-years	29,845	9,215	23,349	400,747
HR (95% CI)	Reference	0.94 (0.77–1.15)	0.93 (0.80–1.07)	**1.13 (1.03–1.25)**
*P* value	Reference	0.569	0.306	**0.014**
**Stroke**				
Events (%)	539 (13.1)	188 (15.0)	451 (14.1)	7,029 (12.8)
Person-years	30,193	9,121	23,424	406,405
HR (95% CI)	Reference	1.11 (0.94–1.30)	1.05 (0.93–1.20)	1.06 (0.97–1.15)
*P* value	Reference	0.241	0.416	0.240
**CVD-related death**				
Events (%)	28 (0.7)	5 (0.4)	13 (0.4)	218 (0.4)
Person-years	32,187	9,854	25,137	434,017
HR (95% CI)	Reference	0.53 (0.20–1.38)	0.58 (0.30–1.12)	0.68 (0.45–1.01)
*P* value	Reference	0.193	0.107	0.056

Statistically significant values are given in bold at *P* < 0.05. HR calculated by Cox proportional hazards regression analysis after adjustments for age, sex, household income, initial body mass index, systolic blood pressure, fasting serum glucose, total cholesterol, aspartate aminotransferase, Charlson comorbidity index, smoking, alcohol consumption, and exercise. Acronyms: CVD, cardiovascular disease; HR, hazard ratio; CI, confidence interval.

### CVD and CHD risk among selected subgroups

**Figure 2:**
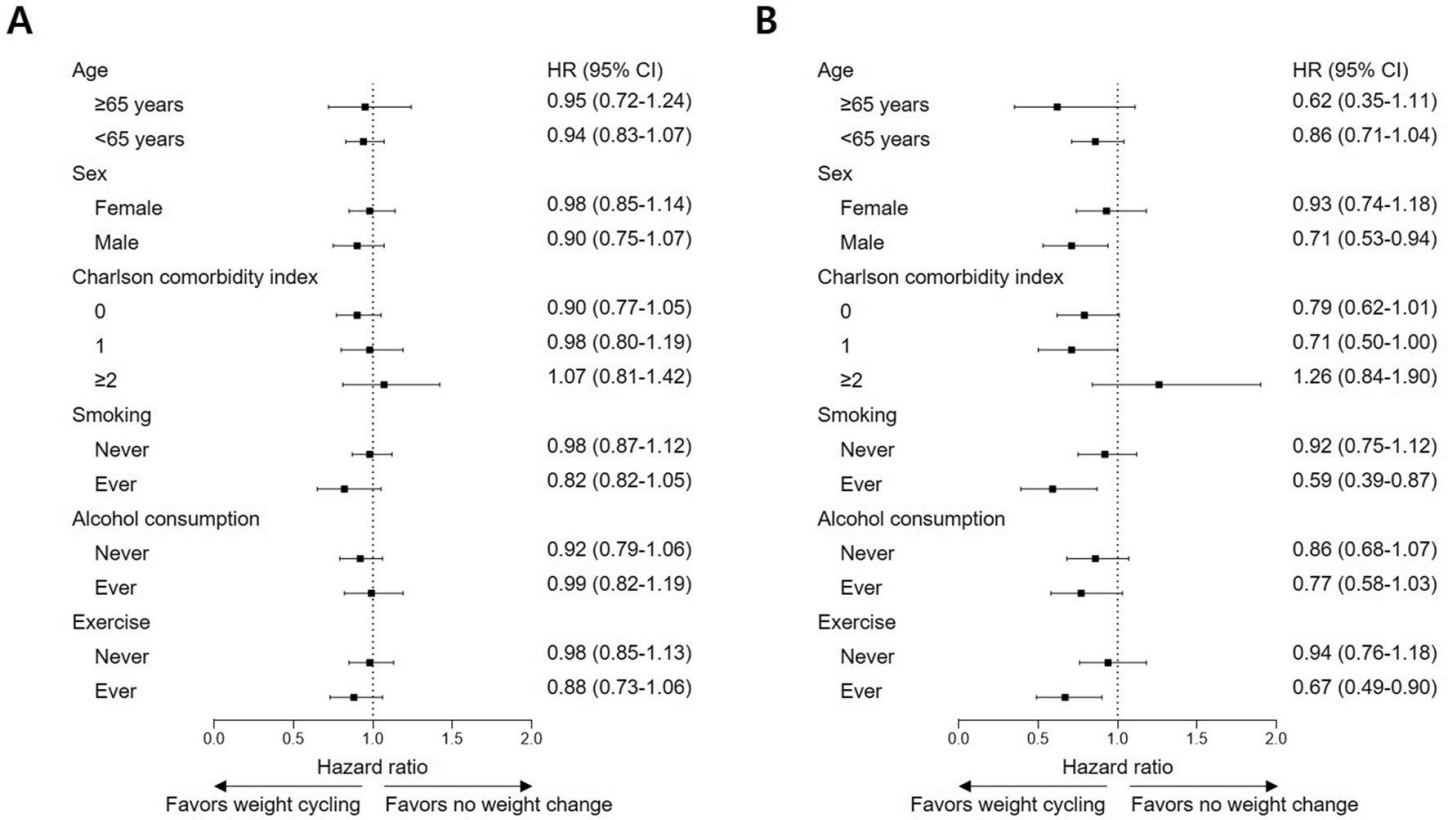
Subgroup analyses on cardiovascular risks of weight cycling compared to no weight change. (**A**) Cardiovascular disease. (**B**) Coronary heart disease.

**Figure 3:**
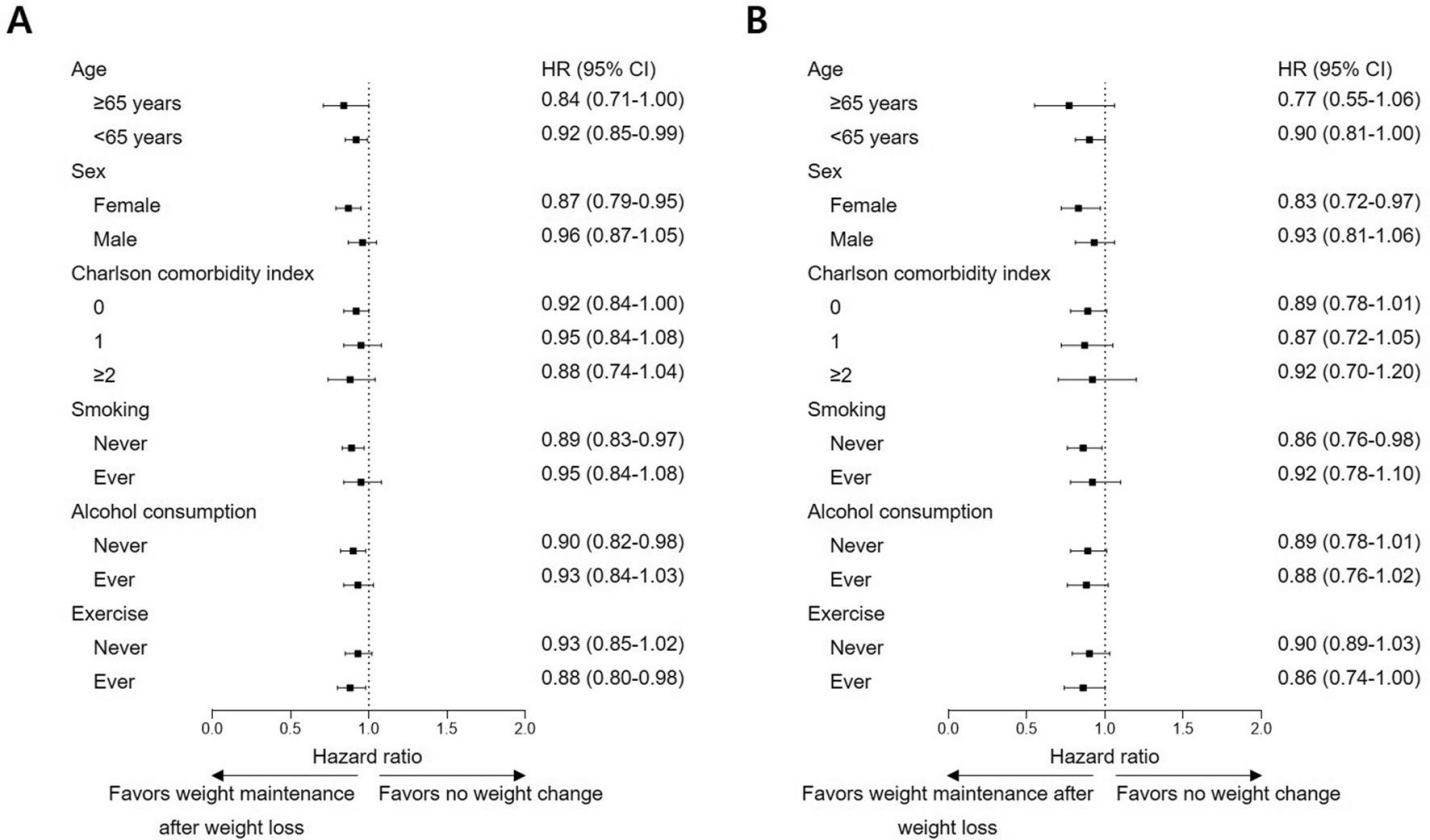
Subgroup analyses on cardiovascular risks of weight maintenance after weight loss compared to no weight change. (**A**) Cardiovascular disease. (**B**) Coronary heart disease.

With intent to clarify whether weight cycling is even worse than no weight change, subgroup analyses were carried out for CVD and CHD. For overall effect on CVD, weight cycling did not significantly increase risk for event compared to no weight change in age, sex, Charlson comorbidity index, smoking, alcohol consumption, and exercise-stratified subgroups (Fig. 2A; Supplementary Table 2). On the contrary, weight cycling was actually beneficial compared to no weight change in male (HR, 0.71; 95% CI, 0.53–0.94; *P* = 0.016), Charlson comorbidity index of 1 (HR, 0.71; 95% CI, 0.50–1.00; *P* = 0.048), ever smoking (HR, 0.59; 95% CI, 0.39–0.87; *P* = 0.008), and ever exercise (HR, 0.67; 95% CI, 0.49–0.90; *P* = 0.008; Fig. 2B; Supplementary Table 3). Confirmations regarding whether weight maintenance after weight loss shows coherent risk reduction against CVD and CHD were followed. In accordance with the primary findings, risk reduction was detected in all subgroups with most of them presenting with statistical significance (Fig. 3A; Supplementary Table 4). Similar results were also found for CHD with slightly smaller number of statistically significant subgroups (Fig. 3B; Supplementary Table 5).

### Effects of no weight change, weight cycling, and weight maintenance after weight loss on change in cardiometabolic risk factors

Changes in systolic and diastolic blood pressure, FSG, TC, and liver function test, including AST, ALT, and γ-GT, were evaluated to ascertain potential impact of BMI maintenance status on cardiometabolic risk factors (Table 4). Among no weight change and weight cycling groups, only the mean change in TC (− 3.3 mg/dL vs − 0.1 mg/dL) was significantly different (*P* = 0.002). However, weight maintenance after weight loss showed significant decreases in systolic (− 3.3 mmHg) and diastolic (− 2.5 mmHg) blood pressures, FSG (− 0.6 mg/dL), TC (− 7.4 mg/dL), AST (− 2.3 IU/L), ALT (− 5.9 IU/L), and γ-GT (− 4.1 IU/L). When compared weight maintenance after weight loss to weight cycling, significantly decreased systolic and diastolic blood pressure, TC, ALT, and γ-GT were noticed. These results suggest that weight maintenance after weight loss significantly ameliorate cardiometabolic risk factors, but the ameliorative effects are offset when regain weight after weight loss.

**Table 4:** Change in blood pressure, fasting glucose, total cholesterol, and liver function at 3rd health examination according to the body mass index maintenance status.

	No weight change	Weight cycling	Weight maintenance after weight loss	*P*_1–2_	*P*_1–3_	*P*_2–3_
Systolic blood pressure, mmHg	− 0.9 (17.8)	− 1.3 (19.1)	− 3.3 (18.3)	0.416	< 0.001	< 0.001
Diastolic blood pressure, mmHg	− 1.4 (12.4)	− 1.2 (13.2)	− 2.5 (12.3)	0.657	< 0.001	0.001
Fasting serum glucose, mg/dL	2.5 (32.3)	0.9 (35.0)	− 0.6 (36.9)	0.078	< 0.001	0.220
Total cholesterol, mg/dL	− 3.3 (36.2)	− 0.1 (39.5)	− 7.4 (38.2)	0.002	< 0.001	< 0.001
Aspartate aminotransferase, IU/L	− 0.9 (19.3)	− 1.9 (30.8)	− 2.3 (20.9)	0.064	< 0.001	0.577
Alanine aminotransferase, IU/L	− 2.0 (25.7)	− 1.2 (32.8)	− 5.9 (26.0)	0.321	< 0.001	< 0.001
γ-glutamyl transpeptidase, IU/L	− 0.1 (41.6)	− 0.4 (46.1)	− 4.1 (48.2)	0.790	< 0.001	0.017

Data are mean (standard deviation). Pooled *P* value calculated by t-test. *P*_1–2_: comparison between maintenance and weight gain after weight loss groups. *P*_1–3_: comparison between maintenance and weight loss maintenance groups. *P*_2–3_: comparison between weight gain after weight loss and weight loss maintenance groups.

## Discussion

In the present study of 67,101 Korean obese participants at baseline, we found that cardiovascular risk is significantly related to the BMI maintenance status. Continuous weight gain was associated with an increased CVD risk, whereas weight loss after weight maintenance and weight maintenance after weight loss was associated with reduced CVD risk. As for CHD, weight maintenance after weight gain was a high-risk group, while weight loss after weight maintenance, weight cycling, and weight maintenance after weight loss were beneficial. In addition, these associations of BMI maintenance status upon CVD risk appeared to change significantly in partial subgroups with distinctive characteristic. To the best of our knowledge, it is the first study to show that BMI maintenance status supports the evidence for evaluation of CVD risk along with clinical characteristics.

While there was no study that extensively studied the association of BMI maintenance status and CVD risk among obese patients according to the BMI change at 3-time frames, some previous studies have explored the effects of BMI on risk of CVD. Karimi et al.^17^ found that low energy density diet-associated weight reduction is related to significant decrease in waist circumference, FSG, TC, low-density lipoprotein cholesterol, and weight regain in individuals with history of recent weight reduction. In the present study, liver function and blood pressure were also ameliorated by the weight maintenance after weight loss. On the contrary, some risk factors, such as diastolic blood pressure and TC, were even less reduced in the weight cycling group compared to the no weight change group. Attenuation of the liver function and blood pressure by weight maintenance after weight loss seem to be managed by improved glucose homeostasis, reduced local inflammation in the liver and heart, decreased lipid levels, enhanced insulin sensitivity within the liver, and attenuated cardiac glucose metabolism as proven in obese mice^18^. These results indicate that cardioprotective effects of the maintenance of weight loss likely to be mediated by improvements in cardiometabolic risk factors, as well as liver function, but the protective effects become offset in weight cycling.

While risk of CHD was significantly attenuated by weight maintenance after weight loss, there was no evidence of risk reduction for stroke. As shown in a recent case–control study conducted from 2015 to 2017, high body mass index was independently associated with CHD^19^. In contrast, risk of stroke for high BMI was significantly mediated by high blood pressure that no significant association is found between high BMI index and stroke after adjustment for systolic blood pressure^20^. Therefore, there may not be an independent risk of being obese for weight maintenance after weight loss to attenuate, but additional weight gain would trigger development of stroke for obese population.

It is important to note that there were 3 significantly beneficial groups compared to no weight change group, including weight cycling. Weight cycling, which refers to a periodic up-and-down weight cycles, is considered to have negative connotations for most people^21^. We also at first hypothesized that weight cycling effect may lead to substantially increased cardiovascular risk for both CHD and stroke in accordance with previous studies^22,23^. Another study also suggested that weight cycling may negatively affect cardiometabolic outcomes compared to stable BMI maintenance^24^. However, a systematic review of literature-based evidences for adverse effects of weight cycling were actually sparse^25^. Collectively, weight cycling may be a potential cardiovascular risk factor for general population compared to no weight change, but losing weight seems to be protective against CHD despite weight regain in obese adults. In addition, the protective effects of the weight cycling varied in subgroups with certain clinical characteristics that it was significantly ameliorative against CHD in male, Charlson comorbidity index of 1, ever smoking, and ever exercise subgroups. In a previous study of CVD risk and lifestyle behaviors, male participants were more physically active and had smoking^26^. In addition, lifestyle behaviors, including dietary habit and physical activity, were significantly dependent to race/ethnic and sex. Apart from lifestyle disparities, sex differences in CVD is considered to be originated from differences in chromosomes and sex steroids hormones that is influenced by pregnancy and the use of exogenous hormone products^27^. To sum up, being male is significantly beneficial from weight cycling against CHD potentially due to primary insusceptibility for CVD, whereas there was no evidence of weight cycling-related protective effect for women.

In conclusion, weight maintenance after weight loss is the most favorable BMI maintenance status from the perspective of cardiovascular health, whereas continuous weight gains and weight maintenance after weight gain increases CVD risk in Korean obese population. Weight cycling was also significantly protective against CHD, but the benefits remained significant only in male, Charlson comorbidity index of 1, ever smoking, and ever exercise subgroups. To sum up, weight loss attempts seem to reduce CVD risk regardless weight cycling.

### Study limitations

Despite some notable strengths of this study, including large study population, wide spectrum of variables, including household income, comorbidities, behavioral factors, and health characteristics, there were several underlying limitations that need to be considered when interpreting our results. The operational definition of CVD is potentially prone to misdiagnosis regarding the low autopsy rate and unusually high CHD among men compared to stroke in South Korea. Although the operational definition applied in the present study has been adopted in many studies using the NHIS-HEALS, more precise definition of CVD is required to validate our findings^28^. In addition, the study population is from a single country. Validations are necessary to generalize the obtained results to population of another race or ethnicity. Lastly, potentially important confounders relevant to associations between BMI maintenance and CVD, such as dietary habits and medications, were not taken into the analyses due to data availability. Future studies need to adopt more comprehensive dataset to validate our results.

## Supplementary Information

Supplementary Information 1.

## Data Availability

The authors have no right to share the database used in this study. To access the database, one should submit the security memorandum and pledge to the Institutional Review Board of Korean National Health Insurance Service. After the approval, data are provided with anonymized personally identifiable information. Any other researchers can access the data in the same manner. Contact information: Tel: + 82 337362432, Website: https://nhiss.nhis.or.kr.
