# Supplementary material for: Association of weight fluctuation with cardiovascular disease risk among initially obese adults
Source: Sci Rep. 2021 May 12;11:10152. doi: 10.1038/s41598-021-89666-7 (PMC8115677; doi:10.1038/s41598-021-89666-7)
Supplement: Supplementary file 1 — Supplementary Information 1. [file 41598_2021_89666_MOESM1_ESM.docx]

**Association of weight fluctuation with cardiovascular disease risk among initially obese adults**

Seogsong Jeong^1^, Seulggie Choi^1^, Jooyoung Chang^1^, Kyuwoong Kim^2^, Sung Min Kim^1^, Seo Yun Hwang^3^, Joung Sik Son^4^, Gyeongsil Lee^5^, Sang Min Park^1,5,*^

1 Department of Biomedical Sciences, Seoul National University College of Medicine, Seoul, Republic of Korea.

2 National Cancer Control Institute, National Cancer Center. Goyang-si, Gyeonggi-do, Republic of Korea.

3 School of Health and Environmental Science, Korea University, Seoul, South Korea.

4 Department of Family Medicine, Korea University Guro Hospital, Seoul 08308, South Korea.

5 Department of Family Medicine, Seoul National University Hospital, Seoul, Republic of Korea.

**
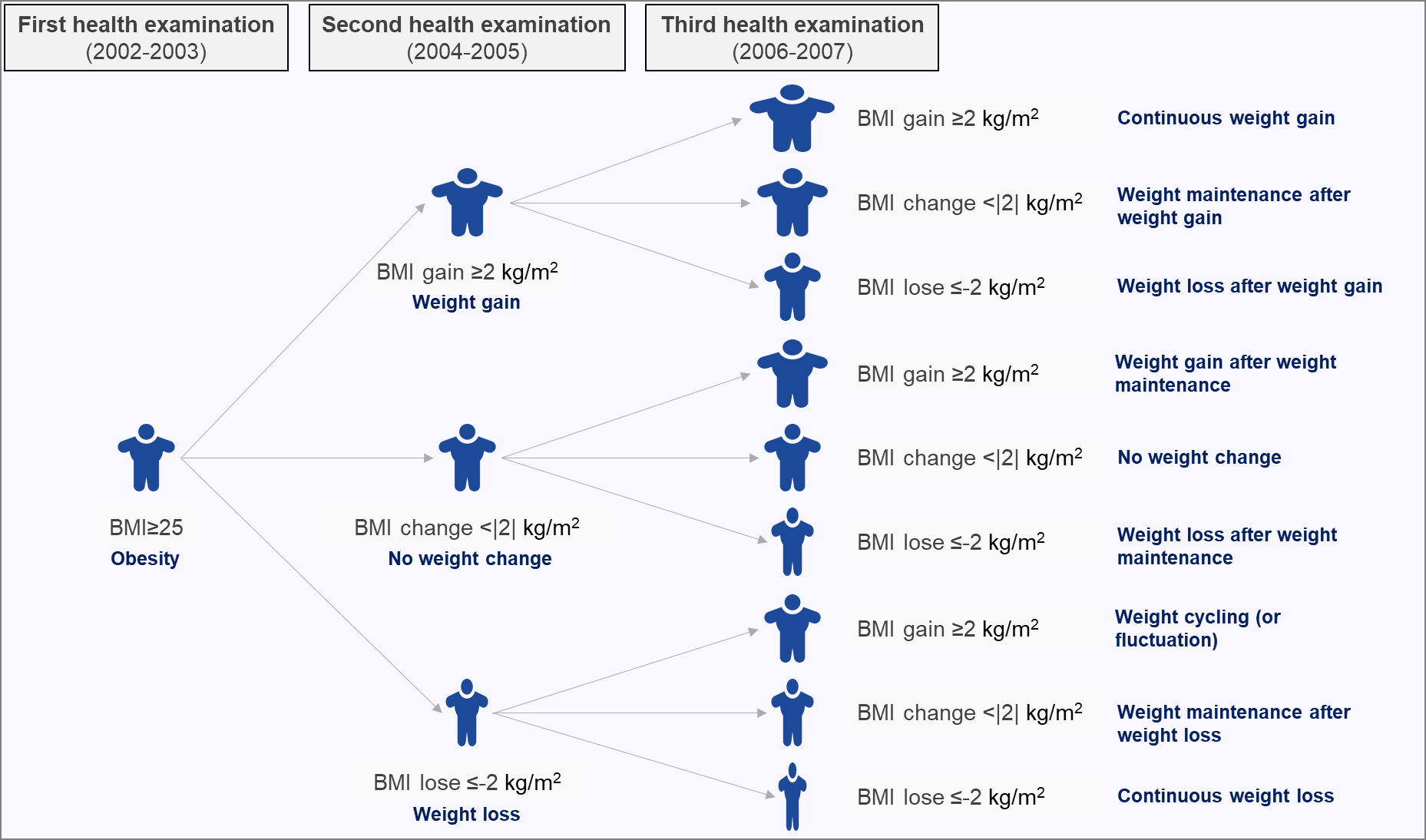
**

**Supplementary Figure 1. Description of 9 body mass index maintenance status categories**

**Supplementary Table 1. Effects of changes in body mass index between first period and second period health examinations on risk of cardiovascular disease, coronary heart disease, stroke, and cardiovascular disease-related death**

| Outcome | Weight gain | No weight change | Weight loss |
| --- | --- | --- | --- |
| CVD |  |  |  |
| Events (%) | 447 (26.9) | 14,325 (23.9) | 1,306 (23.9) |
| Person-years | 11,119 | 412,464 | 37,472 |
| HR (95% CI)^a^ | 1.16 (1.05-1.27) | Reference | 1.00 (0.95-1.06) |
| HR (95% CI)^b^ | 1.10 (1.00-1.21) | Reference | 0.96 (0.91-1.02) |
| HR (95% CI)^c^ | 1.11 (0.98-1.26) | Reference | 0.93 (0.86-1.00) |
| HR (95% CI)^d^ | 1.08 (0.99-1.19) | Reference | 0.92 (0.87-0.98) |
| P value^d^ | 0.095 | Reference | 0.007 |
| CHD |  |  |  |
| Events (%) | 228 (13.7) | 6,685 (11.1) | 570 (10.4) |
| Person-years | 11,832 | 436,687 | 39,772 |
| HR (95% CI)^a^ | 1.26 (1.10-1.44) | Reference | 0.94 (0.86-1.02) |
| HR (95% CI)^b^ | 1.25 (1.10-1.43) | Reference | 0.93 (0.86-1.02) |
| HR (95% CI)^c^ | 1.25 (1.05-1.49) | Reference | 0.86 (0.76-0.97) |
| HR (95% CI)^d^ | 1.23 (1.08-1.41) | Reference | 0.88 (0.81-0.96) |
| P value^d^ | 0.002 | Reference | 0.005 |
| Stroke |  |  |  |
| Events (%) | 223 (13.4) | 7,722 (12.9) | 742 (13.6) |
| Person-years | 12,117 | 442,607 | 40,029 |
| HR (95% CI)^a^ | 1.06 (0.92-1.21) | Reference | 1.06 (0.99-1.15) |
| HR (95% CI)^b^ | 0.97 (0.85-1.10) | Reference | 0.99 (0.92-1.07) |
| HR (95% CI)^c^ | 0.99 (0.84-1.17) | Reference | 1.00 (0.91-1.10) |
| HR (95% CI)^d^ | 0.94 (0.83-1.08) | Reference | 0.97 (0.90-1.05) |
| P value^d^ | 0.387 | Reference | 0.439 |
| CVD-related death |  |  |  |
| Events (%) | 5 (0.3) | 241 (0.4) | 35 (0.6) |
| Person-years | 12,983 | 472,873 | 42,822 |
| HR (95% CI)^a^ | 0.76 (0.31-1.84) | Reference | 1.61 (1.13-2.29) |
| HR (95% CI)^b^ | 0.66 (0.27-1.61) | Reference | 1.45 (1.02-2.07) |
| HR (95% CI)^c^ | 0.45 (0.14-1.41) | Reference | 1.23 (0.81-1.87) |
| HR (95% CI)^d^ | 0.60 (0.25-1.45) | Reference | 1.34 (0.93-1.92) |
| P value^d^ | 0.252 | Reference | 0.118 |

HR calculated by Cox proportional hazards regression analysis. ^a^not adjusted. ^b^adjusted for age and sex. ^c^adjusted for age, sex, household income, initial body mass index, systolic blood pressure, fasting serum glucose, total cholesterol, aspartate aminotransferase, Charlson comorbidity index, smoking, alcohol consumption, and exercise frequency after washing out 3 years of latent period for sensitivity analysis. ^d^adjusted for factors included in Model C. Acronyms: CVD, cardiovascular disease; HR, hazard ratio; CI, confidence interval; CHD, coronary heart disease.

**Supplementary Table 2. Subgroup analyses of cardiovascular disease risk in participants with weight cycling compared to no weight change group**

| Subgroup | No weight change | | Weight cycling | | |
| --- | --- | --- | --- | --- | --- |
|  | Event (%) | HR (95% CI) | Event (%) | HR (95% CI) | P value |
| Age |  |  |  |  |  |
| ≥65 years | 1,777 (46.5) | Reference | 56 (43.4) | 0.95 (0.72-1.24) | 0.685 |
| <65 years | 11,364 (22.2) | Reference | 255 (22.7) | 0.94 (0.83-1.07) | 0.334 |
| Sex |  |  |  |  |  |
| Female | 5,204 (26.6) | Reference | 187 (27.2) | 0.98 (0.85-1.14) | 0.799 |
| Male | 7,937 (22.4) | Reference | 124 (21.9) | 0.90 (0.75-1.07) | 0.232 |
| Charlson comorbidity index |  |  |  |  |  |
| 0 | 7,720 (20.9) | Reference | 161 (20.5) | 0.90 (0.77-1.05) | 0.171 |
| 1 | 3,645 (28.2) | Reference | 100 (30.0) | 0.98 (0.80-1.19) | 0.805 |
| ≥2 | 1,776 (34.1) | Reference | 50 (37.6) | 1.07 (0.81-1.42) | 0.645 |
| Smoking |  |  |  |  |  |
| Never | 8,631 (25.0) | Reference | 244 (26.8) | 0.98 (0.87-1.12) | 0.807 |
| Ever | 4,510 (22.0) | Reference | 67 (19.6) | 0.82 (0.65-1.05) | 0.113 |
| Alcohol consumption |  |  |  |  |  |
| Never | 6,986 (26.0) | Reference | 192 (26.1) | 0.92 (0.79-1.06) | 0.224 |
| Ever | 6,155 (21.9) | Reference | 119 (23.0) | 0.99 (0.82-1.19) | 0.903 |
| Exercise |  |  |  |  |  |
| Never | 6,985 (25.4) | Reference | 196 (27.2) | 0.98 (0.85-1.13) | 0.786 |
| Ever | 6,156 (22.3) | Reference | 115 (21.6) | 0.88 (0.73-1.06) | 0.187 |

Data are HR (95% CI). HR calculated by Cox proportional hazards regression analysis after adjustments for age, sex, household income, initial body mass index, systolic blood pressure, fasting serum glucose, total cholesterol, aspartate aminotransferase, Charlson comorbidity index, smoking, alcohol consumption, and exercise, except for the factor involved in stratification of subgroups. Acronyms: HR, hazard ratio; CI, confidence interval.

**Supplementary Table 3. Subgroup analyses of coronary heart disease risk in participants with weight cycling compared to no weight change group**

| Subgroup | No weight change | | Weight cycling | | |
| --- | --- | --- | --- | --- | --- |
|  | Event (%) | HR (95% CI) | Event (%) | HR (95% CI) | P value |
| Age |  |  |  |  |  |
| ≥65 years | 555 (14.5) | Reference | 12 (9.3) | 0.62 (0.35-1.11) | 0.107 |
| <65 years | 5,635 (11.0) | Reference | 112 (10.0) | 0.86 (0.71-1.04) | 0.110 |
| Sex |  |  |  |  |  |
| Female | 2,188 (11.2) | Reference | 75 (10.9) | 0.93 (0.74-1.18) | 0.553 |
| Male | 4,002 (11.3) | Reference | 49 (8.7) | 0.71 (0.53-0.94) | 0.016 |
| Charlson comorbidity index |  |  |  |  |  |
| 0 | 3,785 (10.3) | Reference | 67 (8.5) | 0.79 (0.62-1.01) | 0.054 |
| 1 | 1,676 (13.0) | Reference | 33 (9.9) | 0.71 (0.50-1.00) | 0.048 |
| ≥2 | 729 (14.0) | Reference | 24 (18.0) | 1.26 (0.84-1.90) | 0.264 |
| Smoking |  |  |  |  |  |
| Never | 3,834 (11.1) | Reference | 99 (10.9) | 0.92 (0.75-1.12) | 0.403 |
| Ever | 2,356 (11.5) | Reference | 25 (7.3) | 0.59 (0.39-0.87) | 0.008 |
| Alcohol consumption |  |  |  |  |  |
| Never | 3,095 (11.5) | Reference | 77 (10.5) | 0.86 (0.68-1.07) | 0.180 |
| Ever | 3,095 (11.0) | Reference | 47 (9.1) | 0.77 (0.58-1.03) | 0.079 |
| Exercise |  |  |  |  |  |
| Never | 3,114 (11.3) | Reference | 81 (11.2) | 0.94 (0.76-1.18) | 0.604 |
| Ever | 3,076 (11.2) | Reference | 43 (8.1) | 0.67 (0.49-0.90) | 0.008 |

Data are HR (95% CI). HR calculated by Cox proportional hazards regression analysis after adjustments for age, sex, household income, initial body mass index, systolic blood pressure, fasting serum glucose, total cholesterol, aspartate aminotransferase, Charlson comorbidity index, smoking, alcohol consumption, and exercise, except for the factor involved in stratification of subgroups. Acronyms: HR, hazard ratio; CI, confidence interval.

**Supplementary Table 4. Subgroup analyses of cardiovascular disease risk in participants with weight maintenance after weight loss compared to no weight change group**

| Subgroup | No weight change | | Weight maintenance after weight loss | | |
| --- | --- | --- | --- | --- | --- |
|  | Event (%) | HR (95% CI) | Event (%) | HR (95% CI) | P value |
| Age |  |  |  |  |  |
| ≥65 years | 1,777 (46.5) | Reference | 150 (41.3) | 0.84 (0.71-1.00) | 0.045 |
| <65 years | 11,364 (22.2) | Reference | 818 (21.8) | 0.92 (0.85-0.99) | 0.018 |
| Sex |  |  |  |  |  |
| Female | 5,204 (26.6) | Reference | 465 (23.9) | 0.87 (0.79-0.95) | 0.003 |
| Male | 7,937 (22.4) | Reference | 503 (23.2) | 0.96 (0.87-1.05) | 0.371 |
| Charlson comorbidity index |  |  |  |  |  |
| 0 | 7,720 (20.9) | Reference | 547 (20.5) | 0.92 (0.84-1.00) | 0.048 |
| 1 | 3,645 (28.2) | Reference | 279 (28.7) | 0.95 (0.84-1.08) | 0.433 |
| ≥2 | 1,776 (34.1) | Reference | 142 (30.6) | 0.88 (0.74-1.04) | 0.136 |
| Smoking |  |  |  |  |  |
| Never | 8,631 (25.0) | Reference | 685 (24.1) | 0.89 (0.83-0.97) | 0.005 |
| Ever | 4,510 (22.0) | Reference | 283 (22.4) | 0.95 (0.84-1.08) | 0.419 |
| Alcohol consumption |  |  |  |  |  |
| Never | 6,986 (26.0) | Reference | 568 (24.7) | 0.90 (0.82-0.98) | 0.016 |
| Ever | 6,155 (21.9) | Reference | 400 (22.1) | 0.93 (0.84-1.03) | 0.154 |
| Exercise |  |  |  |  |  |
| Never | 6,985 (25.4) | Reference | 572 (25.2) | 0.93 (0.85-1.02) | 0.110 |
| Ever | 6,156 (22.3) | Reference | 396 (21.6) | 0.88 (0.80-0.98) | 0.017 |

Data are HR (95% CI). HR calculated by Cox proportional hazards regression analysis after adjustments for age, sex, household income, initial body mass index, systolic blood pressure, fasting serum glucose, total cholesterol, aspartate aminotransferase, Charlson comorbidity index, smoking, alcohol consumption, and exercise, except for the factor involved in stratification of subgroups. Acronyms: HR, hazard ratio; CI, confidence interval.

**Supplementary Table 5. Subgroup analyses of coronary heart disease risk in participants with weight maintenance after weight loss compared to no weight change group**

| Subgroup | No weight change | | Weight maintenance after weight loss | | |
| --- | --- | --- | --- | --- | --- |
|  | Event (%) | HR (95% CI) | Event (%) | HR (95% CI) | P value |
| Age |  |  |  |  |  |
| ≥65 years | 555 (14.5) | Reference | 42 (11.6) | 0.77 (0.55-1.06) | 0.113 |
| <65 years | 5635 (11.0) | Reference | 392 (10.5) | 0.90 (0.81-1.00) | 0.043 |
| Sex |  |  |  |  |  |
| Female | 2188 (11.2) | Reference | 190 (9.8) | 0.83 (0.72-0.97) | 0.017 |
| Male | 4002 (11.3) | Reference | 244 (11.3) | 0.93 (0.81-1.06) | 0.276 |
| Charlson comorbidity index |  |  |  |  |  |
| 0 | 3785 (10.3) | Reference | 256 (9.6) | 0.89 (0.78-1.01) | 0.077 |
| 1 | 1676 (13.0) | Reference | 118 (12.1) | 0.87 (0.72-1.05) | 0.154 |
| ≥2 | 729 (14.0) | Reference | 60 (12.9) | 0.92 (0.70-1.20) | 0.529 |
| Smoking |  |  |  |  |  |
| Never | 3834 (11.1) | Reference | 289 (10.2) | 0.86 (0.76-0.98) | 0.018 |
| Ever | 2356 (11.5) | Reference | 145 (11.5) | 0.92 (0.78-1.10) | 0.809 |
| Alcohol consumption |  |  |  |  |  |
| Never | 3095 (11.5) | Reference | 243 (10.6) | 0.89 (0.78-1.01) | 0.078 |
| Ever | 3095 (11.0) | Reference | 191 (10.6) | 0.88 (0.76-1.02) | 0.099 |
| Exercise |  |  |  |  |  |
| Never | 3114 (11.3) | Reference | 245 (10.8) | 0.90 (0.79-1.03) | 0.132 |
| Ever | 3076 (11.2) | Reference | 189 (10.3) | 0.86 (0.74-1.00) | 0.045 |

Data are HR (95% CI). HR calculated by Cox proportional hazards regression analysis after adjustments for age, sex, household income, initial body mass index, systolic blood pressure, fasting serum glucose, total cholesterol, aspartate aminotransferase, Charlson comorbidity index, smoking, alcohol consumption, and exercise, except for the factor involved in stratification of subgroups. Acronyms: HR, hazard ratio; CI, confidence interval.
